# Contribution of nestin positive esophageal squamous cancer cells on malignant proliferation, apoptosis, and poor prognosis

**DOI:** 10.1186/1475-2867-14-57

**Published:** 2014-06-20

**Authors:** Beilong Zhong, Tao Wang, Xueping Lun, Jinli Zhang, Sannv Zheng, Weilin Yang, Weiqiang Li, Andy Peng Xiang, Zhenguang Chen

**Affiliations:** 1 Department of Thoracic Surgery, the Fifth Affiliated Hospital, Sun Yat-sen University, Zhuhai, Guangdong 519000, China; 2 Center for Stem Cell Biology and Tissue Engineering, Sun Yat-sen University, Key Laboratory for Stem Cells and Tissue Engineering, Ministry of Education, Guangzhou, Guangdong, China; 3 Department of Biochemistry, Zhongshan Medical School, Sun Yat-sen University, Guangzhou, Guangdong, China; 4 Department of Thoracic Surgery, the First Affiliated Hospital, Sun Yat-sen University, Guangzhou, Guangdong 510080, China; 5 Lung Cancer Research Center of Sun Yat-sen University, Guangzhou, Guangdong 510080, China; 6 Department of Cardiothoracic Surgery of East Division, the First Affiliated Hospital, Sun Yat-sen University, Guangzhou, Guangdong 510080, China; 7 Guangzhou Research Institute of Traumatic Surgery, the Fourth Affiliated Hospital, Ji’nan University, Guangzhou, Guangdong 510220, China; 8 Department of Anesthesiology and Operating Room of East Division, the First Affiliated Hospital, Sun Yat-sen University, Guangzhou, Guangdong, China

**Keywords:** Esophagus, Cancer, Esophageal squamous cell carcinoma, Nestin, Intermediate filament, Proliferation, Apoptosis

## Abstract

**Background:**

The stem cell-associated intermediate filament nestin has recently been linked with neoplastic transformation, but the specific mechanism by which nestin positive tumor cells leads to malignant invasion and metastasis behaviors of esophageal squamous cell carcinoma (ESCC) remains unclear.

**Methods:**

To obtain insight into the biological role of nestin in ESCC, we explored the association of the nestin phenotype with malignant proliferation and apoptosis in esophageal squamous cancer cells. Nestin expression was determined in ESCC specimens and cell lines, and correlated with clinicopathological properties, including clinical prognosis and proliferative markers. The association of the nestin phenotype with apoptotic indicators was also analyzed.

**Results:**

Nestin was expressed in ESCC specimens and cell lines. ESCC patients with nestin-positive tumors had significantly shorter median survival and progression-free survival times than those with nestin-negative tumors. Positive staining for the proliferation markers Ki67 and PCNA (proliferating cell nuclear antigen) was detected in 56.9% and 60.2% of ESCC specimens, respectively, and was strongly correlated with the nestin phenotype. Notably, expression of cyclin dependent kinase-5 (CDK5) and P35 was detected in 53.8% and 48.4% of ESCC specimens, respectively, and was strongly associated with the nestin phenotype.

**Conclusion:**

Our data demonstrated nestin expression in ESCC specimens and cell lines, and revealed a strong association of the nestin phenotype with poor prognosis in ESCC patients. Furthermore, we showed that nestin positive ESCC cells played an important role in the malignant proliferation and apoptosis.

## Introduction

Esophageal cancer is a malignancy of the esophagus common throughout the world that is characterized by its high invasiveness and mortality. Histologically, more than 90% of esophageal cancers are either esophageal squamous cell carcinomas (ESCCs) or esophageal adenocarcinomas (EACs); in China, ESCC is the predominant histological subtype and accounts for nearly 90% of all esophageal cancers [1,2]. Despite considerable advancements in surgical techniques and multidisciplinary treatments based on chemotherapy and radiotherapy, the overall 5-year survival rate of ESCC patients has remained low (15%–39%) [3]. The current view is that the failure of ESCC treatments reflects the frequent recurrence and metastasis of this cancer type. These phenomena involve several events, including proliferation and invasion of the primary tumor, sustained lymphangiogenesis, and evasion of apoptosis; of these steps, proliferation of the primary tumor appears to be an integral feature of the molecular and cellular pathogenesis and metastasis of esophageal cancer [4,5].

Nestin is a member of the class VI family of intermediate filament proteins. Nestin was first identified as a protein expressed in progenitor cells of the central and peripheral nervous systems, but has since also been found in other tissues [6,7]. Notably, it has been proposed that nestin may be a proliferative and multipotency potential indicator in several types of progenitor cells [8-13]. Recent reports have linked nestin with malignant characteristics in different cancers and suggested that abundant nestin positive cancer cells correlate with greater malignancy and poor prognosis [14-17]. We previously demonstrated that the majority of tumor cells in non-small cell lung cancer samples are nestin-positive and showed that nestin expression was positively correlated with the subset of lung cancer patients displaying poor outcomes and high levels of proliferative markers [18,19]. Subsequent studies have revealed that nestin knockdown inhibits cell proliferation and G1/S arrest in human non-small cell lung cancer cells, possibly via downregulation of AKT-GSK3β-cyclin D signaling. However, the specific function of nestin positive tumor cells in the invasive and metastatic behaviors of esophageal cancer remains unclear. Thus, the precise mechanisms of nestin positive cancer cells action in the proliferation and metastasis of ESCC require further elucidation.

In the present study, we sought to characterize the nestin phenotype in ESCC of Chinese population and assess its association with esophageal cancer cell proliferative properties and clinical prognosis and pathological parameters. Because no previous studies have provided detailed information on signaling mechanisms involved in the proliferation of esophageal cancer cells relevant to the nestin phenotype, we further investigated the mechanism underlying this linkage.

## Materials and methods

### Tissue specimens

A total of 93 ESCC samples were randomly selected from our tissue database. Samples were obtained from patients treated in the Department of Thoracic Surgery from the First Affiliated Hospital of Sun Yat-sen University between September 2005 and March 2009. The selection criteria included: 1) all cases were confirmed by histopathology; 2) all cases were performed the esophagectomy treatment; 3) at least six lymph nodes were examined for pathological diagnosis; 4) none of the patients had received neoadjuvant chemotherapy or radiotherapy. Clinical information was obtained by reviewing preoperative and perioperative medical records or via telephone or written correspondence. Cases were staged based on the tumor-node-metastasis (TNM) classification of the International Union Against Cancer, revised in 2002 [20,21]. The use of human materials was approved by the Medical Ethical Committee of The First Affiliated Hospital, Sun Yat-sen University (No. 2008–7). Clinical characteristics of patients were shown in Table 1.

**Table 1:** Association of nestin expression with clinicopathological features in ESCC

	**Characteristics**	**No. of patients**	**Nestin expression *n *** **(%)**		*P*-value^*^
			**Positive**	**Negative**	
Gender	Male	72	24 (33.3)	48 (66.7)	0.686
	Female	21	8 (38.1)	13 (61.9)	
Age (y)	**≤**60	51	16 (31.4)	35 (68.6)	0.497
	**>**60	42	16 (38.1)	26 (61.9)	
Differentiation	Poor	32	6 (18.7)	26 (81.3)	0.070
	Moderate	44	19 (43.2)	25 (56.8)	
	Well	17	7 (41.2)	10 (58.8)	
TNM stage	I	5	1 (20)	4 (80)	0.133
	II	56	23 (41.1)	33 (58.9)	
	III + IV	32	9 (30.8)	23 (69.2)	
Lymph nodes	Positive	34	9 (26.5)	25 (73.5)	0.221
	Negative	59	23 (39.0)	36 (61.0)	
Ki67 expression	Positive	53	23 (43.4)	30 (56.6)	0.036
	Negative	40	9 (22.5)	31 (77.5)	
PCNA expression	Positive	56	26 (46.4)	30 (53.6)	0.003
	Negative	37	6 (16.2)	31 (83.8)	
CDK5 expression	Positive	50	23 (46.0)	27 (54.0)	0.022
	Negative	43	10 (23.3)	33 (76.7)	
P35 expression	Positive	45	24 (53.3)	21 (46.7)	0.031
	Negative	48	15 (31.2)	33 (68.8)	

*Chi-square test.

### Cell lines

The esophageal squamous cell cancer cell lines, Eca-109 and TE-1, were obtained from the Cell Bank of the Chinese Academy of Sciences (Shanghai, China), and cultured according to the specific cell bank protocol.

### Immunohistochemical staining

The immunohistochemical procedure was similar to previously reported protocols [18,19,22]. Anti-nestin (AB5922; Millipore, Temecula, CA, USA; 1:500 dilution), anti-Ki-67 (SC-15402; Santa Cruz Biotechnology, Santa Cruz, CA, USA; 1:500 dilution), anti-PCNA (PC10; Cell Signaling Technology, Danvers, MA, USA; 1:4000 dilution), anti-CDK5 (SC-6247; Santa Cruz Biotechnology; 1:1000 dilution), and P35 (SC-820; Santa Cruz Biotechnology; 1:400 dilution) were used as primary antibodies. Ki67 and PCNA staining in ESCC tissue samples was quantified with an optical density-based method using Image-Pro plus 6.0 software (Media Cybernetics, Inc.; Rockville, MD, USA). Double staining of nestin and Ki-67 or nestin and PCNA were processed using strept avidin-biotin complex (SABC) reagent (MXB, KIT-9999, China) and biotinylated antibody was used as secondary antibody [23].

### RT-PCR analysis

Total RNA was extracted, and amplified by reverse transcription-polymerase chain reaction (RT-PCR) using the following primer pairs: nestin, 5’-GAG GAC CAG AGT ATT GTG AGA C-3’ and 5’-CAC AGT GGT GCT TGA GTT TC-3 (368 bp); and β-actin (internal control), 5’-GTG GGG CGC CCC AGG CAC CA-3 and 5-CTC CTT AAT GTC ACG CAC GAT TTC-3’ (540 bp).

### Immunoblotting analysis

Proteins were separated, electrotransferred to polyvinylidene difluoride (PVDF) membranes, blocked with a solution of Tris-buffered saline containing 0.1% Tween-20 (TBST), incubated with mouse anti-nestin antibody (AB5922; Millipore; 1:500 dilution) overnight, and detected with a horseradish peroxidase-conjugated anti-mouse secondary antibody (Cell Signaling Technology). An anti-GAPDH (glyceraldehyde-3-phosphate dehydrogenase) antibody (SC-81545; Santa Cruz Biotechnology) was used as an internal control.

### Statistical analysis

All calculations were performed using SPSS V.14.0 statistical software (Chicago, IL, USA). T-tests, Pearson’s and Spearman's coefficient of correlation, logistic regression, or Chi-square tests were applied as appropriate. Median survival time (MST) and progression-free survival time (PFS) were calculated from the date of surgery until the date of tumor recurrence and last follow-up, and the association of nestin expression with MST and PFS was presented as Kaplan-Meier plots. Univariate and multivariate analyses were performed using Cox proportional hazards regression to determine the prognostic effects of nestin expression and potential clinical variables on MST and PFS.

## Results

### Basic clinical information and follow-up studies

In total, 72 male and 21 female patients with esophageal cancer subjected to curative surgical resection were enrolled in the study. The mean age of patients was 60.96 ± 9.98 years (range, 38 to 86 years). All cases were esophageal squamous cell cancer. Cases were classified as stage I (n = 5), stage II (n = 56), stage III (n = 28) and stage IV (n = 4), and categorized according to differentiation status as poor (n = 32), intermediate (n = 44), and well differentiated (n = 17). Patient data were analyzed after 5-years of follow-up, and information was obtained for 94.6% (88 of 93) of patients. Pathological characteristics of patients are shown in Table 1.

### Nestin expression in ESCC tissue specimens

**Figure 1:**
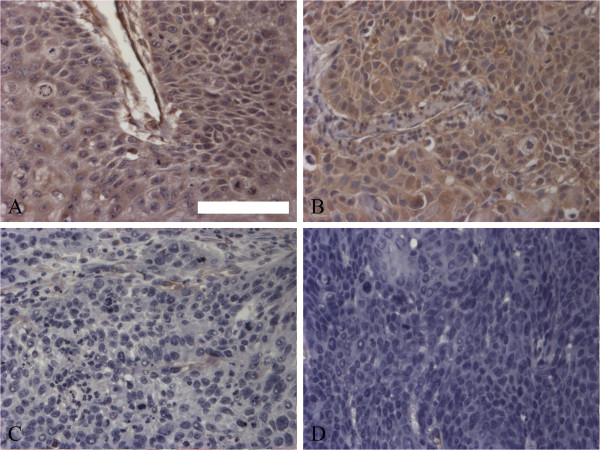
**Nestin expression in the cytoplasm and nucleus of ESCC specimens. (A** and **B)** Nestin-positive staining; **(C** and **D)** weak or negative nestin staining (Scale bar, 100 μm).

Using immunohistochemical scoring methods and optical density statistical software, we estimated nestin antibody staining results qualitatively and quantitatively. Among all tissue specimens, 32 cases (34.4%) were nestin positive (Figure 1A and B) and 61 cases (65.6%) were nestin negative (Figure 1C and D). Regarding to the heterogeneity of nestin staining intensity, the mean optical density was 0.141 (minimum: 0.090; maximum: 0.231) in positive cases and was 0.042 (minimum: 0.002; maximum: 0.089) in negative cases. As shown in Figure 1, nestin expression in tumor cells was detected in both the cytoplasm and nucleus.

### Nestin expression in ESCC cell lines

**Figure 2:**
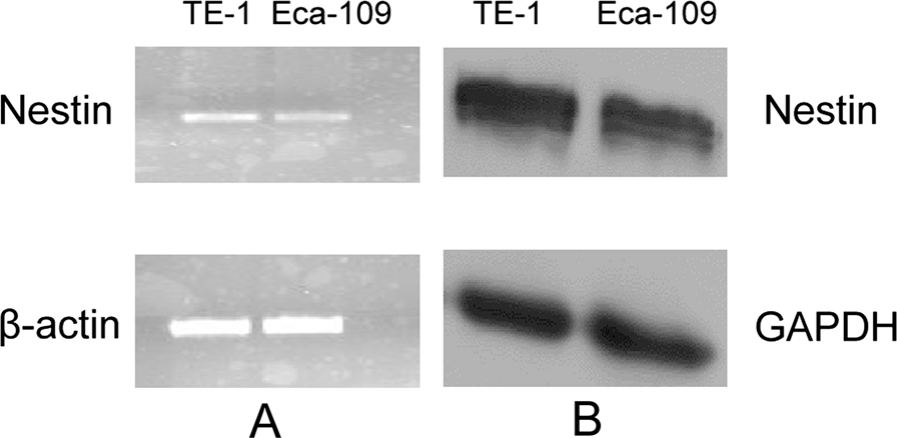
Nestin mRNA and protein expression in Eca-109 and TE-1 ESCC cell lines detected by RT-PCR (A) and immunoblotting analysis (B).

To further examine the expression status of nestin in ESCC, we examined its expression patterns in the ESCC cell lines, Eca-109 and TE-1. Nestin mRNA and protein were detected in both cell lines (Figure 2A and B).

### Association of nestin expression with poor prognosis in ESCC patients

**Figure 3:**
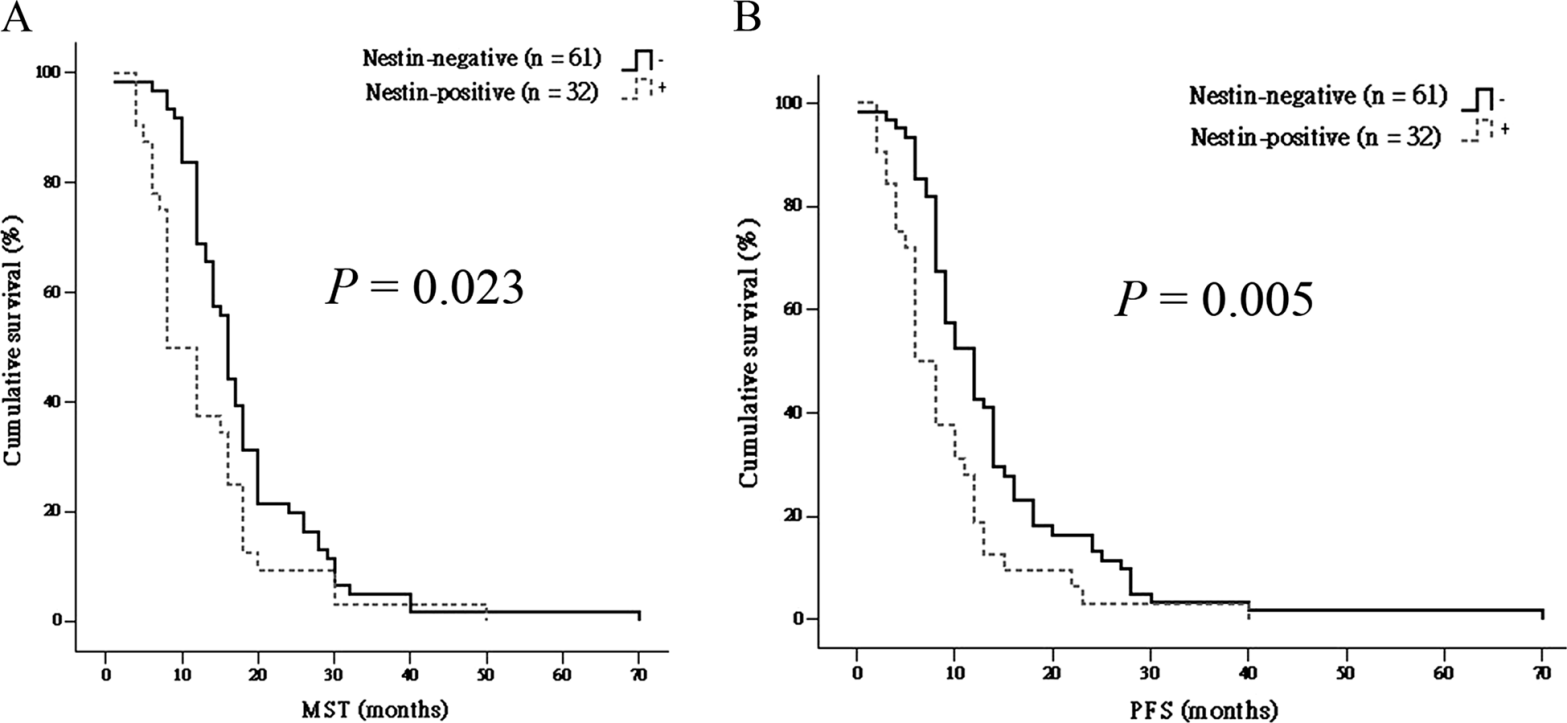
**Kaplan-Meier plot depicting the differences in MST (A) and PFS (B) between nestin-positive and -negative groups, dichotomized based on the median value of nestin expression in tumor lesions.** *P < 0.05 (ANOVA).

The baseline characteristics of the study population with regard to the nestin phenotype and the results of multivariate analyses are presented in Tables 1 and 2, respectively. In the study population as a whole, MST was 15.2 ± 4.2 months (95% CI: 13.3 - 16.7 months). Considering subpopulations based on nestin expression status, the MST of nestin-positive patients (8.3 ± 2.4 months, 95% CI: 5.7 – 10.3 months) was significantly shorter than that of nestin-negative patients (16.0 ± 5.34 months, 95% CI: 14.9 - 17.1 months; *P* = 0.023; Figure 3A). PFS for the entire study population was 12.4 ± 3.9 months (95% CI: 10.4 - 4.4 months). As was the case for MST, PFS was significantly shorter for nestin-positive patients (9.3 ± 3.0 months, 95% CI: 6.6 - 11.9 months) than for nestin-negative patients (14.0 ± 4.1 months, 95% CI: 11.4 - 16.7 months; *P* = 0.005; Figure 3B). A multivariate statistical analysis (Table 2) showed that nestin was a significant prognostic indicator independent of other clinical and pathological factors (HR: 1.586; *P* = 0.047).

**Table 2:** Univariate and multivariate analyses of nestin expression in relation to median survival time of ESCC patients

**Variable**	**Univariate analysis**			**Multivariate analysis**		
	**HR**	**95% CI**	***P-value***	**HR**	**95% CI**	***P-value***
Nestin expression						
Positive	1.579	1.025–2.432	0.038	1.586	1.007–2.499	0.047
Negative	1.000			1.000		
Differentiation						
Poor	0.680	0.442–1.049	0.081	0.658	0.412–1.053	0.080
Moderate and well	1.000			1.000		
TNM classification						
III + IV	1.268	0.820–1.961	0.286	0.862	0.201–3.692	0.842
I + II	1.000			1.000		
Sex						
Male	1.181	0.716–1.946	0.515	1.397	0.826–2.363	0.212
Female	1.000			1.000		
Age						
≥60 years	0.881	0.582–1.334	0.550	0.755	0.486–1.172	0.210
<60 years	1.000			1.000		
Lymph nodes metastasis						
With	1.330	0.864–2.048	0.195	1.870	0.451–7.757	0.388
Without	1.000			1.000		

*Abbreviations:HR* hazard ratio; *LN* lymph nodes.

*Chi-square test.

### Association of nestin with tumor cell proliferative markers

**Figure 4:**
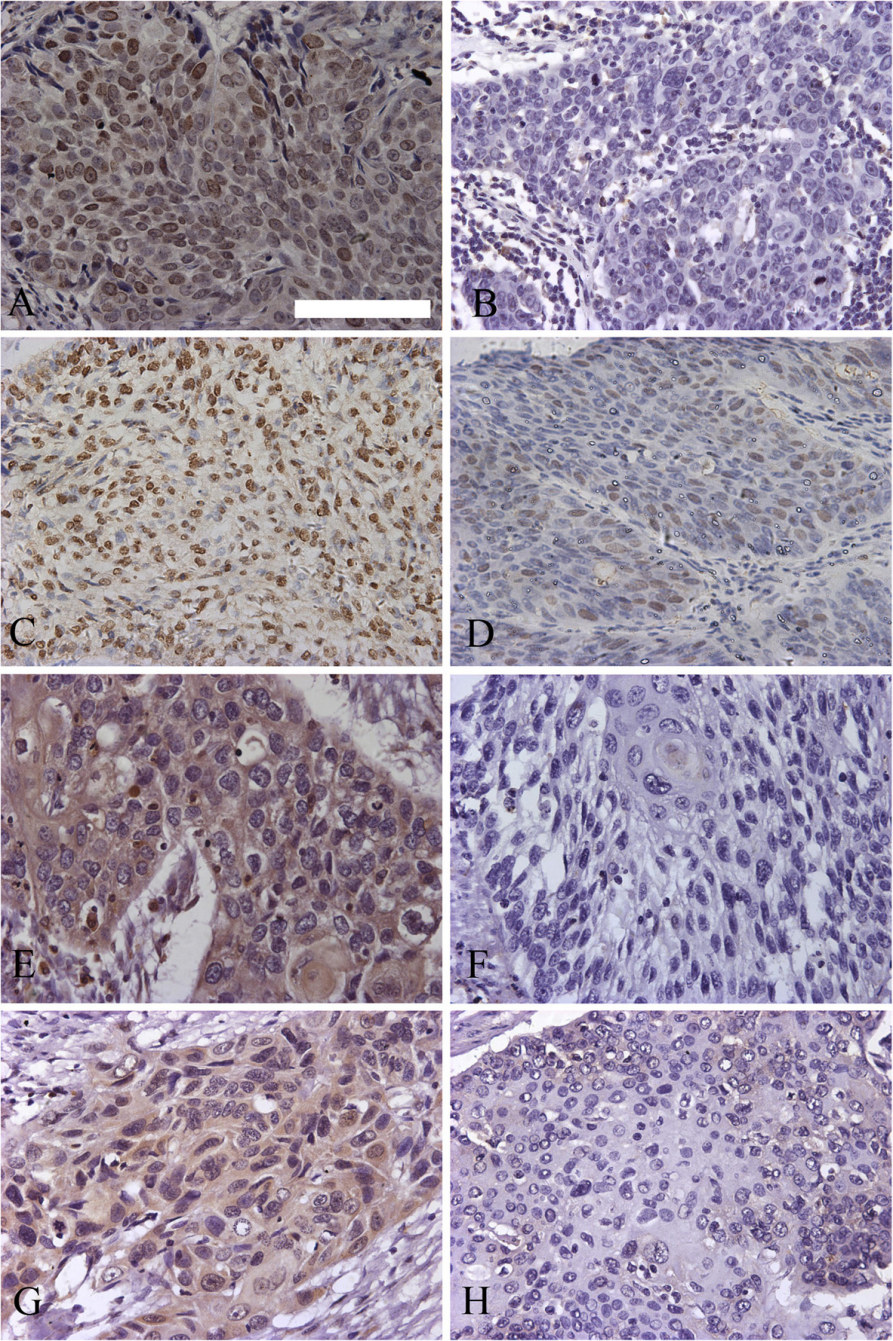
Strong (A) and weak (B) Ki67 staining in ESCC specimens; strong (C) and weak (D) PCNA staining in ESCC specimens; strong (E) and weak (F) CDK5 staining in ESCC specimens; strong (G) and weak (H) P35 staining in ESCC specimens (Scale bar, 100 μm).

**Figure 5:**
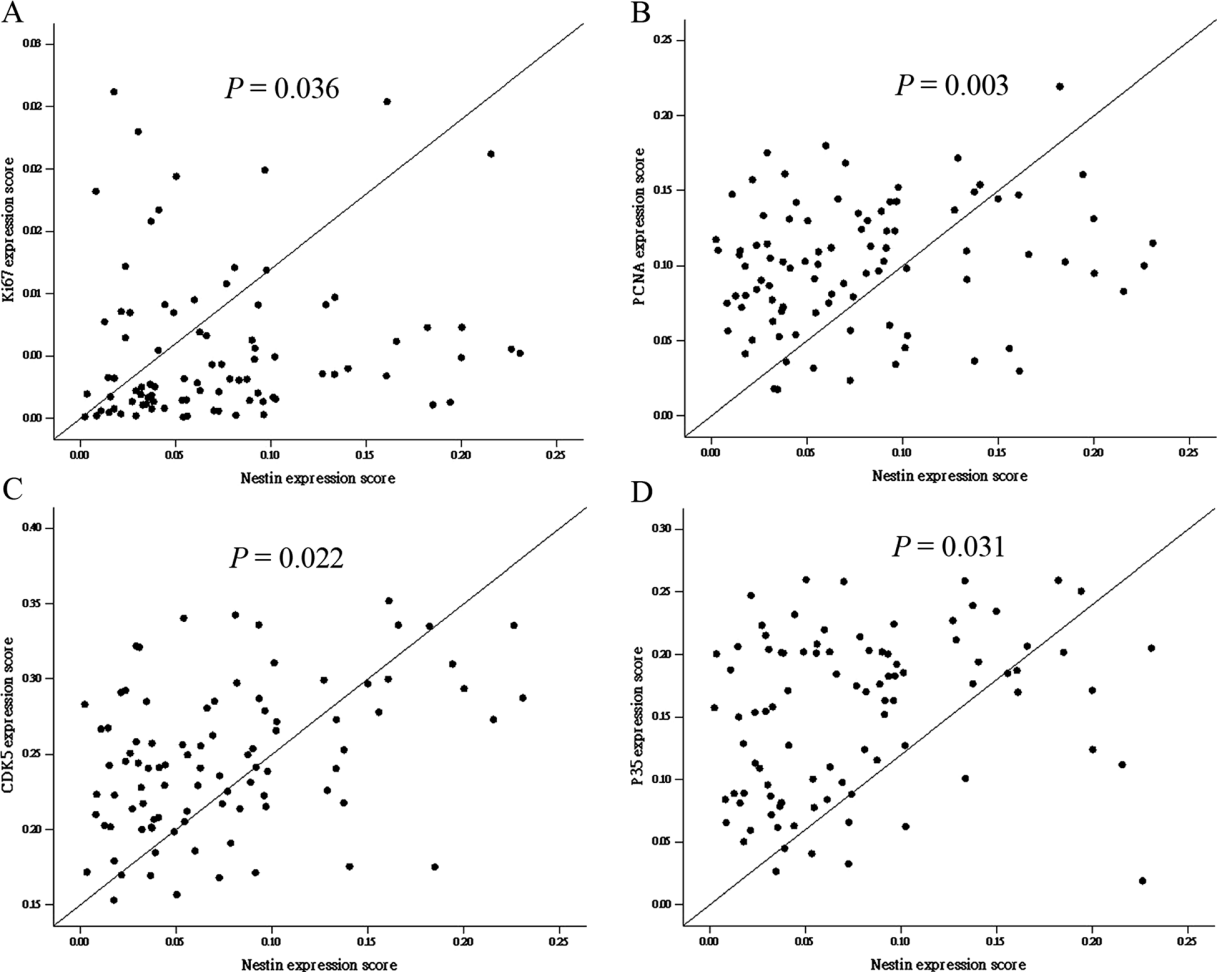
**Significant correlation of nestin expression levels with (A) Ki67 expression levels (*r*** **= 0.223; *P*** **< 0.05), (B) PCNA expression levels (*r*** **= 0.328; *P*** **< 0.05), (C) CDK5 expression levels (*r*** **= 0.240; *P*** **< 0.05), and (D) P35 expression levels (*r*** **= 0.223261; *P*** **< 0.05).** Each point represents one ESCC specimen.

**Figure 6:**
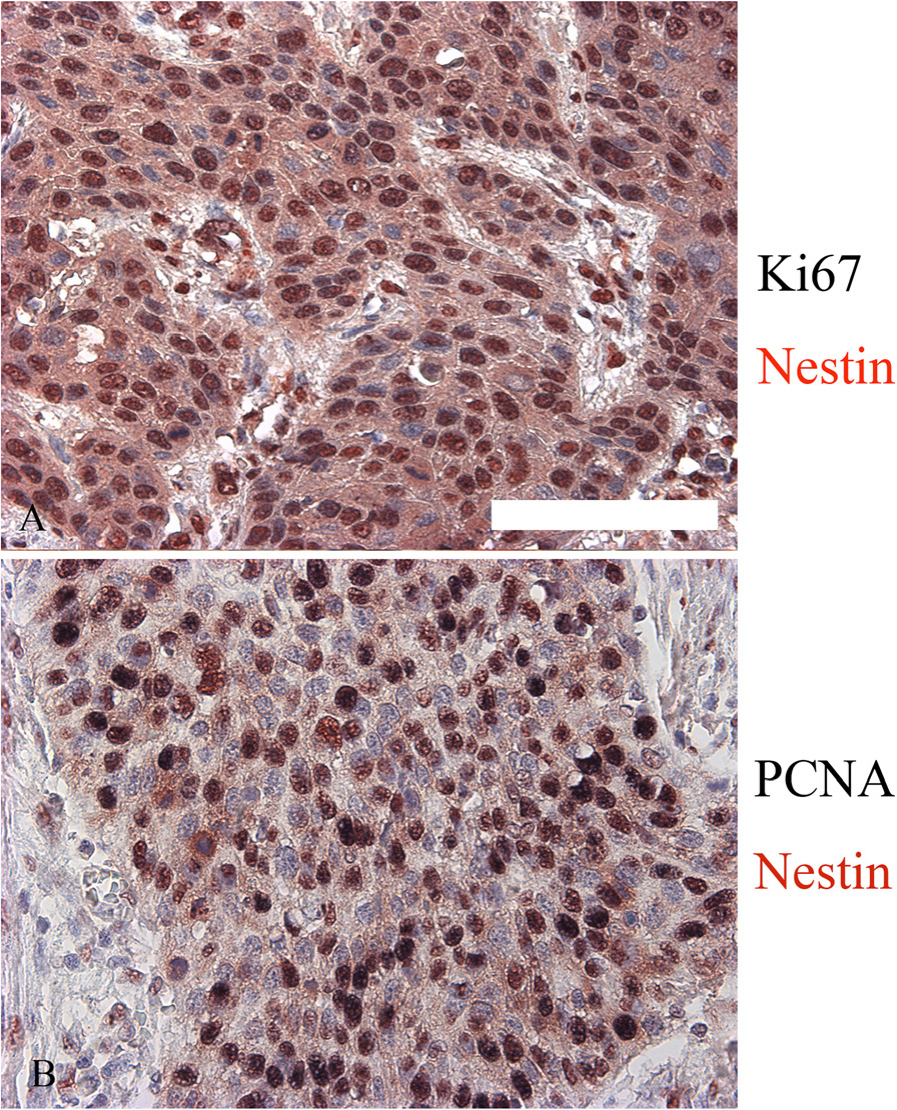
**Significant double staining of nestin and Ki67 was shown in A and B.** Red color indicated nestin staining and black color indicated Ki67 staining. Meanwhile, strong double staining of nestin and PCNA was shown in C and D. Red color indicated nestin staining and black color indicated PCNA staining (Scale bar, 100 μm).

Expression of the proliferative markers Ki67 and PCNA in ESCC tissue samples was determined by immunohistochemical staining. Of the 93 cases of ESCC, 53 (56.9%) were positive for the expression of Ki67, which was mainly nuclear (Figure 4A), and 40 (43.1%) were negative for Ki67 expression (Figure 4B). Similar results were obtained for the expression of PCNA; in 56 cases (60.2%), cells were positive for PCNA expression (Figure 4C) and in 37 cases (43.1%), cells were negative for PCNA expression (Figure 4D). As expected, PCNA expression was mainly detected in ESCC nuclei (Figure 4C). Ki67 and PCNA expression was quantified (Table 3) using an optical density scoring method employing image analysis software (see Materials and Methods). As shown in Figure 5 (A and B) and Table 1, a subsequent Pearson’s correlation analysis revealed a significant relationship between the nestin phenotype and Ki67 and PCNA optical density (Ki67: *r* = 0.223, *P* = 0.036; PCNA: *r* = 0.328, *P* = 0.003). As shown in Figure 6, double staining of nestin and Ki-67 or nestin and PCNA was performed and revealed the status of proliferation of nestin-positive cells.

**Table 3:** Association of nestin expression with Ki67, PCNA, CDK5 and P35 expression, determined using an optical density method

	**Nestin-positive**	**Nestin-negative**	*P*-value^*^
Ki67	0.0124 ± 0.0033	0.0057 ± 0.0010	<0.0001
PCNA	0.1318 ± 0.0060	0.0831 ± 0.0052	<0.001
CDK5	0.2609 ± 0.0120	0.2140 ± 0.0053	<0.001
P35	0.2050 ± 0.0118	0.1478 ± 0.0100	<0.0001

*Chi-square test.

### Association of nestin with tumor cell apoptotic markers

As a first step toward identifying the signaling pathway underlying the nestin phenotype, we assessed the expression of P35 and CDK5 (cyclin-dependent kinase 5), which is regulated by P35, in ESCC specimens. Of the 93 samples, 50 (53.8%) were positive for CDK5 and 45 (48.4%) were positive for P35 (Figure 4). CDK5 and P35 expression were detected in both nuclei and cytoplasm of ESCC cells. As shown in Figure 5, an analysis of the relationship between CDK5 and P35 expression, quantified using an optical density-based scoring method (Table 3), and the nestin phenotype revealed a significant Pearson’s correlation coefficient (CDK5: *r* = 0.240, *P* = 0.022; P35: *r* = 0.261, *P* = 0.031).

## Discussion

Nestin was initially discovered based on its expression in neural progenitor cells, where it was considered a marker for distinguishing precursor cells from differentiated cells [24,25]. Subsequent reports have shown that nestin is expressed in breast, prostate and pancreatic cancer, and is positively correlated with tumor malignancy [15,26,27]. Sustained expression of nestin in spindle cells or epithelial cells of esophageal leiomyoma has also been reported [28]. However, no studies have examined nestin expression in ESCC.

Here, we first established that nestin is expressed in 34.4% of ESCC samples in Chinese population, a result similar to that reported for lung squamous cell carcinoma (35.5%) and our previous findings in NSCLC [19,29]. Consistent with these findings, we also detected nestin mRNA and protein in the ESCC cell lines, Eca-109 and TE-1.

Secondly, we showed that the nestin phenotype is significantly associated with poor prognosis in Chinese patients with ESCC. Some studies have reported that nestin expression in vascular sarcoma, pancreatic cancer, and gastrointestinal stromal tumors is closely correlated with the degree of malignancy and patient prognosis [14,15,30]. In our study, we found significant differences in both MST and PFS between patients in nestin-positive and nestin-negative groups. Furthermore, a multivariate statistical analysis showed that nestin was a significant prognostic indicator independent of other clinical and pathological factors. Therefore, our experiments showed that Chinese ESCC patients with nestin-positive tumors had a worse prognosis than those in the nestin-negative group.

Thirdly, we found that the nestin phenotype of ESCC cells was closely related to malignant proliferation. Recent studies have reported that tumor cells with high expression of nestin show rapid proliferation [31]. In our experiments, we used Ki67 and PCNA as proliferative markers. Ki67, a nuclear protein that has been widely used in tumor biology as a marker of tumor-cell proliferation, plays an important role in regulating cell differentiation [32]. PCNA, a trimer that forms a clamp around DNA and promotes DNA polymerase-dependent DNA replication, is highly expressed in proliferating cells, especially tumor cells; in the latter, high expression of PCNA is correlated with high malignancy [33-35]. Among ESCC clinical specimens, 56.9% and 60.2% were positive for Ki67 and PCNA, respectively. Moreover, a quantitative analysis revealed that Ki67 and PCNA expression were positively correlated with nestin expression. Taken together, these findings indicate that the nestin phenotype is positively correlated with ESCC cell proliferation, providing preliminary evidence for a role for nestin in regulating ESCC cell proliferation.

We also investigated possible association of nestin with tumor cells apoptosis by examining the expression of CDK5 and P35 using immunohistochemistry. CDK5 is a member of the cyclin-dependent kinase family that is preferentially expressed in terminally differentiated cells, such as neurons, muscle, and lens fibers [36-40]. CDK5 activity is mainly regulated by its association with P35 (which is often, but not exclusively, associated with neural tissues) and to lesser degree by P39 [40]. CDK5 is able to perform substrate phosphorylation in different cellular compartments, including the cytoplasm and nucleus [41,42]. Some reports suggest that CDK5 may have a significant role in the regulation of breast, lung, and prostate cancer cell proliferation, apoptosis, migration, and invasion [43-45]. We found that 53.8% and 48.4% of ESCC cases were positive for the expression of CDK5 and P35 expression, respectively; CDK5 and P35 were detected in both nuclei and cytoplasm, consistent with a previous report [46]. Importantly, CDK5 and P35 expression were positively correlated with nestin expression, suggesting the possibility that nestin promotes ESCC cell apoptosis. In this latter context, it is conceivable that nestin might serve as a nuclear scaffold for CDK5 that could affect the organization, stability, and activity of the CDK5 and P35 signaling complex, as suggested by the reported importance of nuclear localization in the activity of Cdk5 [42,47].

Based on the data that the nestin phenotype is closely correlated with ESCC cell proliferation and apoptosis, by extension, our results suggest an association with metastasis, consistent with recent reports that tumor cells with high expression of nestin more readily metastasize. Metastasis is a gradual and complex process that requires a collection of dispersed tumor cells with several capabilities, including invasion into the circulatory system from the primary tumor, survival in the circulation, exudation at a distant site, and proliferation in an alien and inhospitable environment [47,48]. The ability of tumor cells to migrate and invade into the circulatory system from the primary tumor is related to tumor cell motility. Cell motility is important during embryonic development as well as in physiological processes of many adult organs. This kind of motility is regulated by numerous mechanisms, including a link between the epithelial cell membrane and the cytoskeleton. One such mechanism is that mediated by intermediate filaments. For example, cytoskeletal reorganization of actin filaments is very important for cell motility and also underlies most cell migration [47,48]. As a member of the class VI family of intermediate filament proteins and a cytoskeletal element, nestin has an effect in tumor cells similar to that of actin. When tumor cells successfully invade the circulatory system from the original tumor and reach an alien immune-surveillance environment, their ability to survive—largely a reflection of their ability to proliferate—becomes the key to their successful metastasis in this environment. In our experiments, nestin expression in ESCC samples was positively correlated with Ki67 and PCNA expression, predicting that nestin-positive esophageal squamous cells would proliferate more rapidly than nestin-negative cells. We also found that MST and PFS of nestin-positive ESCC patients were shorter than those of nestin-negative patients. The current view is that the primary reasons for the failure of ESCC surgical treatments are recurrence and metastasis [4]. Our preliminary studies demonstrating that nestin expression is positively correlated with the proliferative and apoptotic behavior of ESCC provide a possible explanation for the prognostic value of nestin. Collectively, our findings demonstrate that nestin-expressing tumor cells are important for proliferation, apoptosis, migration, and metastasis in ESCC.

While our results suggest that nestin contributes to the proliferation and apoptosis in ESCC cells, other aspects of metastasis, such as angiogenesis, lymphangiogenesis and tumor metabolism, were not directly examined. However, in view of our findings, the role of nestin needs to be considered in clinical diagnosis and as a potential new target for cancer therapy.

In summary, we observed nestin-positive tumor cells in a portion of ESCC samples in Chinese population and demonstrated a significant association of nestin expression with the subset of Chinese ESCC patients displaying poor outcomes and high levels of proliferative markers. Our preliminary findings of positive correlations between nestin expression and CDK5 and P35 expression further support the tentative conclusion that nestin likely promotes ESCC cell apoptosis. Collectively, our data suggest that tumor cells expressing nestin promote ESCC cell proliferation and apoptosis, which may constitute a key mechanism of nestin-mediated metastasis in esophageal cancer cells. Targeted regulation of nestin may thus have therapeutic applications in the treatment of human esophageal cancer.

## Abbreviations

CDK5: Cyclin dependent kinase-5; EAC: Esophageal adenocarcinoma; EC: Esophageal cancer; ESCC: Esophageal squamous cell carcinoma; MST: Median survival time; PCNA: Proliferating cell nuclear antigen; PFS: Progression-free survival; TNM: Tumor-node-metastasis.

## Competing interests

All authors have full access to all of the data in the study and have final responsibility for the decision to submit for publication. The authors have no conflict of interest.

## Author contributions

BZ, TW, and ZC conceived the study, participated in analysis of NSCLC specimens and cell lines, and drafted the manuscript. BZ, TW, JZ, and WY performed histopathological analysis of tumor samples and RT-PCR assay. BZ, XL, and SZ participated in the patient inclusion and manuscript preparation. TW, WY, and WL conducted Western blot analysis and the cell proliferation assay. BZ and ZC performed statistical analysis of all data. APX and ZC designed the experiments, coordinated the study, and drafted the manuscript. All authors have read and approved the final version of the manuscript.
